# Simplified ART Delivery Models Are Needed for the Next Phase of Scale Up

**DOI:** 10.1371/journal.pmed.1001060

**Published:** 2011-07-19

**Authors:** Nathan Ford, Edward J. Mills

**Affiliations:** 1 Médecins Sans Frontières, Geneva, Switzerland; 2 Centre for Infectious Disease Epidemiology and Research, University of Cape Town, Cape Town, South Africa; 3 Faculty of Health Sciences, University of Ottawa, Ottawa, Ontario, Canada

## Abstract

Nathan Ford and Edward Mills discuss broader issues in HIV care delivery raised by research carried out by Lawrence Long and colleagues into down-shifting HIV care to primary health facilities.

**Linked Research Article**

This Perspective discusses the following new study published in *PLoS Medicine*:

Long L, Brennan A, Fox MP, Ndibongo B, Jaffray I, et al. (2011) Treatment Outcomes and Cost-Effectiveness of Shifting Management of Stable ART Patients to Nurses in South Africa: An Observational Cohort. PLoS Med 7(7): e1001055. doi:10.1371/journal.pmed. 1001055

Lawrence Long and colleagues report that “down-referring” stable HIV patients from a doctor-managed, hospital-based ART clinic to a nurse-managed primary health facility provides good health outcomes and cost-effective treatment for patients.

## Decentralized ART Provision a Necessity

Efforts to scale up antiretroviral therapy (ART) for people living with HIV/AIDS in resource-limited settings began with a clear recognition of the need to adapt the model of care from individualized care to a public health approach [1]. Ten years ago, the main model for ART delivery was the Western model: specialized and individualized, with patients receiving careful clinical monitoring and drug regimens that were frequently altered according to tolerability, emergence of resistance, and patient preference. Such a level of care was clearly beyond the capacity of hospital services in sub-Saharan Africa 10 years ago, and which remain for the most part poorly funded, poorly equipped, understaffed, and overwhelmed.

Acknowledging the urgency of scaling up treatment for millions of patients in clinical need, innovative approaches to simplified ART delivery were implemented in parallel with (not subsequent to) formal epidemiological assessments. So, the first randomized trial comparing doctors and nurses in the delivery of ART [2] was published 3 years after such “task shifting” was promoted by the World Health Organization [3]. Whilst trial data were important to validate the task shifting approach, the lack of sufficient numbers of doctors in high-burden countries meant that by the time the evidence was published, hundreds of thousands of patients were already dependent on nurses and other mid-level health workers for their HIV care.

New evidence is welcomed. A study published this week in *PLoS Medicine* by Lawrence Long and colleagues [4] reports the feasibility and benefits of “down-referring” ART patients from hospitals to health centers in South Africa, and shows that ART provision at the health centers was beneficial for both patients (better survival and retention in case) and providers (reduced costs). This type of study is important to stimulate donors and program managers to go further in supporting ART provision beyond centralized, hospital-based facilities. That this type of care is urgently needed is supported by a recent survey by Médecins Sans Frontières revealing that less than one-fifth of public health facilities in the Central African Republic, Guinea, Kenya, Mozambique, and Uganda provided ART [5].

In common with task shifting, which out of necessity was implemented before formal evidence of efficacy was reported, the decentralized provision of ART beyond the hospital level has been happening for several years in many resource-limited settings, where hospitals are few and distant from patients, out of necessity. In Malawi, patients have received ART at health centers since 2006, with outcomes better than those obtained in the hospital [6]. Reports from South Africa [7] and Lesotho [8] show similarly reassuring outcomes for decentralized care.

## Moving ART Provision beyond the Health System

The effectiveness of ART delivery in reducing mortality in resource-limited settings has been established [9], and there is increasing evidence that providing ART earlier will reduce hospitalizations and opportunistic infections [8] and contribute to reducing HIV transmission [10]. But the effectiveness of the health system in delivering ART is questionable, considering the high rates of patient attrition along the treatment cascade from HIV diagnosis to long-term treatment [11]. Given that there are still some 10 million people considered eligible for ART but not receiving it, a strategy of initiating ART treatment for patients in hospitals and then down-referring them to a clinic once they are stable has in many settings already reached its limits.

An important question raised by Long et al. 's study, therefore, is how can research help define future HIV programs, rather than validate what is already happening? Few would disagree that the proper role of a hospital is to treat critically ill patients, not to dispense medicines to healthy people. Yet there is little agreement and no policy guidance about how far we can go beyond the health system. The fact that retention in care is consistently reported to be better at decentralized sites [4],[6],[7],[12] indicates the importance of providing care as close as possible to people's homes.

We need to go much further. The ambition today is to provide ART to many more people, and much earlier in their infection, over a long-term period. Realizing this ambition will depend on defining models of ART delivery that are minimally intrusive to patient's lives. Several studies have demonstrated the feasibility of home-based [13] and community-based [14] ART management, with positive results. Future research on ART delivery should build on these findings in order to help develop the elements that promote early HIV diagnosis, ensuring rapid enrollment into care, and support continuous adherence to an effective treatment regimen such that HIV care is largely a self-managed chronic disease, with the role of hospitals limited to providing care for a sick minority.

## Abbreviations

ART: antiretroviral therapy

## References

[1] Harries AD, Nyangulu DS, Hargreaves NJ, Kaluwa O, Salaniponi FM (2001). Preventing antiretroviral anarchy in sub-Saharan Africa.. Lancet.

[2] Sanne I, Orrell C, Fox MP, Conradie F, Ive P (2010). Nurse versus doctor management of HIV-infected patients receiving antiretroviral therapy (CIPRA-SA): a randomised non-inferiority trial.. Lancet.

[3] World Health Organization (2007). Task shifting: global recommendations and guidelines.. http://www.who.int/healthsystems/task_shifting/en/.

[4] Long L, Brennan A, Fox MP, Ndibongo B, Jaffray I (2011). Treatment outcomes and cost-effectiveness of shifting management of stable ART patients to nurses in South Africa: an observational cohort.. PLoS Med.

[5] Médecins Sans Frontières (2011). Getting ahead of the wave: lessons for the next decade of the AIDS response.. http://www.doctorswithoutborders.org/publications/article.cfm?id=5294&cat=special-report.

[6] Bemelmans M, Van Den Akker T, Ford N, Philips M, Zachariah R (2010). Providing universal access to antiretroviral therapy in Thyolo, Malawi through task shifting and decentralization of HIV/AIDS care.. Trop Med Int Health.

[7] Fatti G, Grimwood A, Bock P (2010). Better antiretroviral therapy outcomes at primary healthcare facilities: an evaluation of three tiers of ART services in four South African provinces.. PLoS ONE.

[8] Ford N, Kranzer K, Hilderbrand K, Jouquet G, Goemaere E (2010). Early initiation of antiretroviral therapy and associated reduction in mortality and morbidity and defaulting in a nurse-managed, community cohort in Lesotho.. AIDS.

[9] The Antiretroviral Therapy in Lower Income Countries (ART-LINC) Collaboration and ART Cohort Collaboration (ART-CC) groups (2006). Mortality of HIV-1-infected patients in the first year of antiretroviral therapy: comparison between low-income and high-income countries.. Lancet.

[10] HIV Prevention Trials Network (12 May 2011) Initiation of antiretroviral treatment protects uninfected sexual partners from HIV infection (HPTN study 052) [press release]. Available: http://www.hptn.org/web%20documents/PressReleases/HPTN052PressReleaseFINAL5_12_118am.pdf. Accessed 15 June 2011

[11] Micek MA, Gimbel-Sherr K, Baptista AJ, Matediana E, Montoya P (2009). Loss to follow-up of adults in public HIV care systems in central Mozambique: identifying obstacles to treatment.. J Acquir Immune Defic Syndr.

[12] Bedelu M, Ford N, Hilderbrand K, Reuter H (2007). Implementing antiretroviral therapy in rural communities: the Lusikisiki model of decentralized HIV/AIDS care.. J Infect Dis.

[13] Jaffar S, Amuron B, Foster S, Birungi J, Levin J (2009). Rates of virological failure in patients treated in a home-based versus a facility-based HIV-care model in Jinja, southeast Uganda: a cluster-randomised equivalence trial.. Lancet.

[14] Decroo T, Telfer B, Biot M, Maïkéré J, Dezembro S (2011). Distribution of antiretroviral treatment through self-forming groups of patients in Tete province, Mozambique.. J Acquir Immune Defic Syndr.

